# Experiences of COVID-19 Patients With Person-Centred Care During Cohort Isolation: A Mixed-Method Study

**DOI:** 10.7759/cureus.78470

**Published:** 2025-02-03

**Authors:** Vera P Van Druten, Andrea D Rozema, Tessel H.G. Hendriksen, Esther de Vries, Dike van de Mheen, Carolina J.P.W. Keijsers, Babette C van der Zwaard, Aukelien Scheffelaar, Lenny M.W. Nahar-van Venrooij

**Affiliations:** 1 Tranzo Scientific Center for Care and Wellbeing, Tilburg School of Social and Behavioral Sciences, Tilburg University, Tilburg, NLD; 2 Jeroen Bosch Academy Research, Jeroen Bosch Hospital, 's-Hertogenbosch, NLD; 3 Department of Medical Psychology, Jeroen Bosch Hospital, 's-Hertogenbosch, NLD; 4 Department of Geriatric Medicine, Jeroen Bosch Hospital, 's-Hertogenbosch, NLD; 5 Department of Orthopaedic Surgery, Jeroen Bosch Hospital, 's-Hertogenbosch, NLD

**Keywords:** care experiences, cohort isolation, covid-19, isolation precautions, person-centred care

## Abstract

Introduction

Quality of care may have been compromised during the exceptionally hectic period of the COVID-19 waves, including isolation precautions.

Aims

Experiences with person-centred care and wellbeing of COVID-19 patients admitted to a cohort isolation nursing ward during the first pandemic wave were explored, including differences between the acute phase (March 2020) and semi-acute phase (April-June 2020).

Methods

A convergent parallel mixed-method design was used. Adult no post-ICU patients visiting the outpatient clinic for COVID-19 aftercare between May and September 2020 six to eight weeks after discharge from the COVID-19 hospital ward were asked to participate. Part 1 comprised Patient Reported Experience Measure (PREM) questionnaires (n=134) and part 2 semi-structured interviews (n=30). Thematic analysis was performed, and themes were linked to the domains of the person-centred care framework.

Results

Part 1: Median scores on all the questions of the PREM were high. The lowest scores on the PREM questionnaire were seen on shared decision-making during the acute phase.

Part 2: In line with this, the interviews also indicated that the majority of patients had positive experiences regarding person-centred care and that patients felt a limited need for interaction in shared decision-making. Additionally, it was learned from the interviews that the domain care environment was affected most by the pandemic: the isolation precautions had an impact on patients’ wellbeing, although they understood the necessity for isolation precautions to protect others. Patients felt lonely, missed physical contact with family and friends, and felt that contact with healthcare professionals was influenced by isolation clothes. Although some still felt isolated, the cohort department contributed to a feeling of engagement with other patients having the same illness.

Conclusion

Delivered care was positively experienced by patients despite the hectic outbreak period. Patients understood and adapted their preferences to the circumstances. However, patients’ wellbeing was influenced by the circumstances in which care was received. To improve patients’ wellbeing during isolation, recognisability of healthcare workers should be stimulated, visits from family and friends should be allowed, and no use of single-room isolation is advised.

## Introduction

In March 2020, the COVID-19 outbreak was declared as a pandemic by the World Health Organization (WHO) [1]. In the Netherlands, patients with suspected or confirmed severe acute respiratory syndrome coronavirus-2 (SARS-CoV-2) infections were quarantined at home or - when medical care was necessary - admitted to isolation wards in the hospital [2]. We were interested in the effects of hospital care in isolation on person-centred care and on patients' wellbeing.

Person-centred care is a widely used approach in healthcare in which the person is seen as an individual with unique values, preferences and needs [3,4]. This approach appears to be useful to establish an interconnected relationship between patients and care professionals and is defined as mutual trust between patients and care professional, knowing the person, their values, biography, and relationships with others, and seeing beyond the personal needs and authenticity [5]. Person-centred care implies providing care in a respectful manner and being responsive to patients’ individual preferences as possible. McCormack and McCance (2006) developed a framework for person-centred nursing used by practitioners to guide decision-making and to enhance engagements with their patients [3]. This framework includes prerequisites (focussing on the nursing attributes) the care environment (focussing on the context for delivered care), and person-centred processes and expected outcomes (as a result of effective person-centred care) [3].

Unfortunately, isolation precautions may have a negative impact on patients’ person-centred care experiences [5-8]. In earlier research, patients reported suboptimal patient-healthcare worker contact with isolation masks blocking facial expressions [6], failures of support, dissatisfaction about care [7], no respect for their needs and preferences, and poor care coordination [8].

Isolation precautions also may have a negative impact on patients’ wellbeing. For example, they cause mental health problems, such as depression, psychological distress, fear, insomnia, and anxiety [9-11]. Furthermore, patients missed (physical) contact with their loved ones [9,12] and felt distanced from the outside world [9].

Due to the high number of admissions to the hospitals during the COVID-19 pandemic, patients received care grouped together in cohort isolation. This means that doors can be left open and staff does not have to change clothes at each door. A room can be for one or more patients and patients can leave the room at any time to go for a walk or exercise or else. Earlier research with cancer patients in cohort isolation showed that patients mainly preferred a multiple-bed environment because the fellow patients’ company provided essential support for patients [13]. Experiences with person-centred care in cohort isolation in these exceptional circumstances were not studied before. We aimed to investigate these experiences. Therefore, we used the person-centred nursing framework to assess person-centred care experiences and wellbeing of COVID-19 patients receiving care provided under strict isolation precautions during the exceptional COVID-19 period. Using a mixed-method design, the qualitative results explained the quantitative results in more depth. Moreover, we hypothesised that more control over the situation by healthcare providers in the hospital results in more effective person-centred care. Therefore, we compared the patients’ experiences admitted during the acute and semi-acute phases of the first wave of the COVID-19 pandemic in the Netherlands.

## Materials and methods

Study design

We used a convergent parallel mixed-method design to develop a complete understanding of the COVID-19 care experiences following the methodology of Creswell et al. [14]. This methodology consists of quantitative and qualitative data collection followed by their separate analysis. Quantitative data were collected by questionnaires (Part 1) and qualitative data (Part 2) by semi-structured interview; both strands were collected at the same time during the same phase of the research process. For data analysis, the strands were kept separate, and prioritised equally. In the results section, the results are described separately. Afterwards, they are integrated and discussed.

Patients were admitted to the hospital between March and June 2020 because of COVID-19 infection in the first pandemic wave in the Netherlands. Starting from May until September 2020, patients visited the COVID-19 aftercare outpatient clinic for check-up, and in this time period, the data collection was done. The patients were asked once to fill out the questionnaire and were interviewed once during the study. After September 2020, we started data analysis. During the analysis, we found that the first wave comprised two separate phases in the hospital (acute and semi-acute). Therefore, the data was split and also studied separately in the acute and semi-acute phases during the analysis depending on the patients’ admission dates: admitted during March 2020, the acute phase, and April-June 2020, the semi-acute phase.

Setting

Eleven emergency hospitals located in the province North Brabant (in the south of the Netherlands, 2.5 million inhabitants) provided COVID-19 care during the first pandemic wave. One top clinical teaching hospital was under research in this study (630 clinical beds, catchment area of 360,000 inhabitants); this hospital was one of those hardest hit. At the peak of the outbreak at the end of March 2020, approximately 70 COVID-19 patients were admitted to the hospital, in cohort isolation on the established COVID-19 hospital ward. From April onwards, the admission rates in the Netherlands decreased [15,16]. More information and experiences with the disease course became present. The day with most hospital admissions in the Netherlands cuts off the acute and semi-acute phases (March 31, 2020, included in the semi-acute phase) [15]. In May 2020, the outpatient clinic for COVID-19 aftercare for patients’ follow-up six to eight weeks after discharge was established from where patients were identified for our study. Patients that were admitted to the ICU had their own aftercare follow-up and were not included in our study.

Participants

We included all patients visiting the outpatient clinic for COVID-19 aftercare who were 18 years and older, no post-ICU patients, length of hospital stay at minimum one day in cohort isolation at the COVID-19 hospital ward, and Dutch speaking. Exclusion criteria were (1) ICU admission, (2) diagnosis of dementia, (3) intellectual disability (if their memory was too limited to answer questions).

Part 1 was obligatory; Part 2 was optional. Purposive sampling was applied for inclusion in the interview with preference for patients with length of stay at a minimum of two days without transfer to another hospital.

Isolation precautions

After being diagnosed, patients were admitted to the COVID-19 nursing ward in a single or multiple room in cohort isolation depending on the severity of the disease. Room doors were left open, patients and healthcare professionals could move around freely. Staff wore hair nets, latex gloves, mouth-nose-masks (FFP2), goggles and protective clothing. Visitors were not allowed.

Part 1: Questionnaires

The applied Dutch Patient Reported Experience Measure (PREM) questionnaire was developed for administration to elicit patients’ experiences with specialised medical care in hospitals according to the following steps [17]. Based on earlier measures [18,19], healthcare providers and insurance companies defined important quality dimensions: information provision, shared decision-making, medication, aftercare, behaviour, cooperation and continuity. The PREM was pre-tested in focus groups among patients, healthcare professionals and providers held by Nivel in 2016 (Nivel is the independent Netherlands Institute for Health Services Research contributing to the effectiveness and quality of the Dutch healthcare system). In the focus groups, these quality dimensions were prioritised and two dimensions were added: healthcare professionals’ competency and waiting times. Next, one question per dimension was formulated, resulting in the PREM for specialised medical care. In the next phase, patient interviews were conducted to test comprehensibility, interpretation and completeness of the questionnaire, resulting in a short and comprehensible questionnaire (described in a national report [17]). Afterwards, face and content validity were concluded to be adequate. In 2017, this PREM was adapted by the Dutch Patient Federation for hospital benchmarking (www.zorgkaartnederland.nl) and became part of daily practice for patients and hospitals to compare providers and professionals. The PREM comprises 13 questions (Table 1); questions 1-7 reflect care experiences (score options; 1-10, or the category ‘not applicable’ (NA)) [20]. Question 8 concerns patients’ healthcare provider recommendation. Question 9 was not analysed because this was part of the interviews (see Part 2). Questions 10-12 concerned patient characteristics. Question 13 concerns self-reported health (score options; bad, moderate, good). The PREM was filled out once on paper directly after their aftercare appointment taking 10-15 minutes.

**Table 1:** PREM questionnaire Patient Reported Experience Measure (PREM) questionnaire for medical specialised care (from Patiëntenfederatie Nederland, Argo: Vragenlijst PREM (Patient Reported Experience Measures) Medisch Specialistische Zorg. Zorgverzekeraars Nederland, Zeist; 2019. https://www.zn.nl/dossier/patientervaringsmetingen/metingen/).

	Questions
Question 1	Did the healthcare worker listen carefully?
Question 2	Was the healthcare worker’s explanation understandable?
Question 3	Did you have confidence in the healthcare worker’s expertise?
Question 4	Were the benefits and disadvantages of the treatment explained?
Question 5	Did you decide together with the healthcare worker which care or treatment you will receive?
Question 6	Was there good cooperation between the care providers in the hospital?
Question 7	How do you judge the (provisional) effect of your treatment?
Question 8	Would you recommend this healthcare provider to other patients with the same disease or health issues?
Question 9	What are you most satisfied with in terms of care? What do you think could be done better in health provision?
Question 10	What is your age?
Question 11	What is your gender?
Question 12	What is your highest level of completed education?
Question 13	In general, how would you describe your health?Note! This question is about the time you were hospitalised because you had Covid-19.

Analysis for Part 1

The patient characteristics such as age and length of hospital stay were presented by mean or median and their deviation measures were distribution-dependent. The patient characteristics such as sex, transfer to another hospital and educational level were presented by frequencies and percentages. Because of expected skewed data for PREM scores [21], categories were made for questions 1-7 in consensus with the research team: scores of 1-5 were interpreted as inadequate, 6-7 as adequate, 8-10 as good experience with care, along with the category NA. To assess the differences between subgroups, for numeric data, the independent t-test or Mann-Whitney U test was applied. For categorical data, the Fisher's exact test or chi-square test (more than two categories) was used [22]. Data were analysed using IBM SPSS Statistics version 25 [23]. A p-value <0.05 was regarded as statistically significant.

Part 2: Semi-structured interviews

An interview guide was developed and pilot-tested twice. After pilot testing, three questions were rephrased and one question about care outcomes was added. No further changes were made during the interviews. The guide comprised 18 questions about patient’s values, preferences [4] and their experiences with person-centred care [3] including four domains: (1) healthcare professional’s abilities, (2) care environment, (3) person-centred processes and (4) results of care (see Appendix table). Thirty interviews were conducted by the first author (VvD) by telephone and were audio recorded. Each patient was interviewed once. Based on literature, data saturation was expected with 12-15 interviews per phase (acute/semi-acute) (i.e., no new information emerging) [24].

Analysis for part 2

Interviews were transcribed verbatim. Transcripts were coded and analysed using Atlas.ti (version 8.4.5). To determine inter-rater reliability, the first six interviews were independently coded by two authors (VvD, LN-vV). On comparing the codes, the agreement between the two authors was acceptable and therefore the remaining interviews were coded by the first author only (VvD). Next, the last author (LN-vV) double-checked all codes to verify if the quotes and codes fitted well. In case of disagreement, a discussion among the research team followed (VvD, AR, AS, LN-vV). Thematic analysis was conducted for identifying, analysing and reporting the data [25]. The steps used were: (1) familiarising yourself with the data, (2) generating initial codes, (3) searching themes, (4) reviewing themes and (5) defining and naming themes [25]. As a result, the data was divided into codes, subthemes and themes, which were afterwards assigned to one of the four domains of the person-centred nursing framework [3]. Similarities and differences between the phases (acute/semi-acute) were investigated per theme, subtheme and code level.

Integration of results: parts 1 and 2

After a separate analysis of parts 1 and 2, the results were integrated [14]. The scores on each question of the PREM were compared with the resulting themes from the interviews. Convergence, divergence and contradictions were investigated and are described in the Discussion section. The person-centred nursing framework was used as guidance to integrate, interpret and discuss the results.

Ethical considerations

The study was conducted in accordance with current laws, public regulations and principles of the Declaration of Helsinki. Approval for local feasibility was given by the review board from the Jeroen Bosch Academy (JBH), the research department of the Jeroen Bosch Hospital, and ethical approval was granted by the METC Brabant (Tilburg, the Netherlands, study number NW2020-42). METC Brabant is a recognised independent committee that reviews research proposals regarding the law for scientific research involving human subjects (WMO) for the JBH after the JBH has given approval for the local feasibility of the research. All patients received written and verbal information about the study and that participation was voluntary, anonymous and they could leave the study at any time. Informed consent was given verbally by all the patients. Verbal informed consent was approved by METC Brabant. Data were handled confidentially and pseudonymised: names and other identifying information were replaced by a code.

## Results

Part 1: questionnaire

Patient Characteristics

In total, 423 patients were admitted to the COVID-19 nursing ward: 70 patients were excluded because they were admitted to the intensive care unit (ICU), 95 patients died during or after admission, and 47 patients were not followed-up in the COVID-19 aftercare outpatient clinic because they were living in other areas or were followed-up by their general practitioner. Then, 211 patients visited the COVID-19 aftercare outpatient clinic (Keijsers et al., 2022), out of which 55 patients were not eligible for inclusion or not invited (the researcher was not always available to visit all the patients because too many patients were visiting the outpatient clinic simultaneously). Of the remaining 156 patients, 138 patients consented to participate. Four patients did not complete the PREM questionnaire, leaving us with 134 patients for analysis (62% male; mean age, 63 years; median length of hospital stay, three days; transfer to another hospital, 27.6%) (see Table 2 for patients’ characteristics).

**Table 2:** Characteristics of patients that visited the COVID-19 aftercare outpatient clinic Note: m = male; SD = standard deviation; IQR = interquartile range; min = minimum score; max = maximum score; PREM = Patient Reported Experience Measure; Q = question; n = number; P = probability; t = t-value; z = z-value; df = degrees of freedom; na = not applicable. The group of patients for the interviews (n=30) is a subgroup of the patients who responded to the PREM questionnaire (n=134). Educational level Low: primary education, prevocational secondary education, first three years of senior general secondary education, first three years of pre-university education and senior secondary vocational education level 1. Middle: four or five years of senior general secondary education, four, five or six years of pre-university education and senior secondary vocational education level 2, 3 or 4. High: higher vocational education and university education. ^1^For 29 patients, their admission overlapped the two phases and they were not counted in the acute and semi-acute phases. ^2^For 23 patients, their hospital admission overlapped the two phases; these patients were only included in the total group analysis. ^3^For four patients, their hospital admission overlapped the two phases; these patients were only included in the total group analysis. ^4^Fisher's exact test. ^5^Independent t-test. ^6^Due to bed shortages, more patients were transferred to other hospitals during the acute phase resulting in a shorter length of stay in this specific hospital. In the semi-acute phase, more beds were available and fewer transfers were conducted and thereby length of stay was longer. ^7^Mann-Whitney U test. ^8^Chi-square test.

						Part 1 Questionnaires					Part2 Interviews				
	Total (n=211)	Acute phase (March 2020) (n=92)^1^	Semi-acute phase (April-June 2020) (n=90)^1^	p	Test statistics	Total (n=134)	Acute phase (March 2020) (n=59)^2^	Semi-acute phase (April-June 2020) (n=52)^2^	p	Test statistics	Total (n=30)	Acute phase (March 2020) (n=13)^3^	Semi-acute phase (April-June 2020) (n=13)^3^	p	Test statistics
	n (% )	n (%)	n (%)			n (%)	n (%)	n (%)			n (%)	n (%)	n (%)		
Sex (m)	131 (62.1)	66 (71.7)	48 (53.3)	0.014^4^	na	86 (64.2)	46 (78.0)	28 (53.8)	0.009^4^	na	20 (66.7)	10 (76.9)	8 (61.5)	0.673^4^	na
Age (years) (mean ± SD)	64.9 ± 13.0	65.6 ± 12.2	63.5 ± 13.9	0.284^5^	t (df) = 1.076 (108)	64.0 ± 12.1	66.0 ± 10.0	60.6 ± 13.9	0.018^5^	t (df) = 403 (109)	60.6 ± 10.1	63.8 ± 12.7	57.4 ± 7.1	0.131^5^	t (df) = 1.579 (18.761)
Transfer to another hospital^6^	44 (20.9)	35 (38)	5 (5.6)	<0.001^4^	na	37 (27.6)	29 (49.2)	5 (9.6)	<0.001^4^	na	1 (3.3)	1 (7.7)	0	1.000^4^	na
Length of hospital stay (days)^6^ (median (IQR))	3 (5)	2 (3)	3 (5)	0.006^7^	Z = -2.741	3 (5)	2 (2)	4 (6)	0.001^7^	Z = -4.217	5 (4)	4 (4)	5 (5)	0.585^7^	Z = - 0.545
Educational level	na	na	na	na	Low	57 (43.8)	26 (44.8)	21 (41.2)	0.117^8^	X^2 ^(df) =4.294 (2)	7 (24.1)	5 (38.5)	2 (15.4)	0.407^8^	X^2 ^(df) =1.797 (2)
					Middle	37 (28.5)	12 (20.7)	19 (37.3)			12 (41.4)	4 (30.8)	5 (38.5)		
					High	36 (27.7)	21 (34.5)	11 (21.6)			10 (34.5)	4 (30.8)	6 (46.2)		
					Missing	4	1	1			1	0	0		
PREM Q13: self-reported health	na	na	na	na	Bad	81 (60.4)	38 (64.4)	27 (51.9)	0.375^8^	X^2^ (df)= 1.961 (2)	21 (70)	7 (53.8)	10 (76.9)	0.421^8^	X^2 ^(df) = 1.729 (2)
					Moderate	34 (25.4)	13 (22.0)	17 (32.7)			5 (15.7)	3 (23.1)	2 (15.4)		
					Good	19 (14.2)	8 (13.6)	18 (15.4)			4 (13.3)	3 (23.1)	1 (7.7)		

Scores from the PREM Questionnaire

The median scores for questions 1-7 were high (Table 3). However, for questions 4 and 5 about shared decision-making, the scores ranged from 1 to 10; 13 patients (9.7%) and 26 patients (19.4%) scored between 1 and 5, respectively, representing an inadequate experience with care.

**Table 3:** Results of PREM questions 1-7. Note: PREM = Patient Reported Experience Measure, IQR = interquartile range, min = minimum score, max = maximum score, n = number, Q = question. ^1^None of these scores between the acute and semi-acute phases differed significantly (Mann-Whitney U-test, all p>0.05).

	Total (n =134)	Acute phase (March 2020) (n=59)	Semi-acute phase (April-June 2020) (n=52)^1^
	Median (IQR) (min; max)	Median (IQR) (min; max)	Median (IQR) (min; max)
Q1	9 (2) (6;10)	9 (2) (6;10)	9 (2) (6;10)
Q2	9 (2) (5;10)	9 (2) (5;10)	9 (2) (5;10)
Q3	9 (1) (6;10)	9 (2) (7;10)	9 (2) (6;10)
Q4	9 (3) (1;10)	8 (4) (1:10)	9 (2) (1;10)
Q5	8 (3) (1;10)	8 (4) (1;10)	8 (2) (1;10)
Q6	9 (2) (4;10)	9 (1) (5;10)	9 (2) (4;10)
Q7	9 (2) (5;10)	9 (2) (7;10)	9 (2) (6;10)

Part 2: interviews

Patient Characteristics

Out of the 76 patients who gave informed consent for a telephone interview, 30 patients were interviewed. The interviews lasted on average 57 (range 34-97) minutes. The interview questions are provided in the Appendix table. Purposive sampling resulted in including patients with a minimum of two days to a maximum of eight days of hospital stay in the acute phase and one transfer to another hospital and for the semi-acute phase two to 15 days of hospital stay and no transfer (Table 2).

Interview results per domain of person-centred care

The identified themes, subthemes, summary of interview data and quotes are shown in Table 4.

**Table 4:** Identified themes and subthemes from the interview data related to domains of person-centred care.

Domain	Theme	Subtheme	Summary of interview data per theme	Quote
Healthcare professional’s abilities	Communication	About treatment	Patients were satisfied regarding the communication with healthcare professionals; their questions about treatment or care were answered; healthcare professionals were friendly.	“They knew it wasn’t pleasant to give me the news, but they did in a very kind way, and it was very clear” [Patient 2, male, age 72 years, three days of admission at the hospital].
		About transfer to another hospital	The information provided about the transfer was clear and correct (although the message was not always pleasant).	
		About discharge	The information provided about discharge was clear and correct.	
		Missed information	A few patients experienced the communication about treatment, transfer to another hospital or information at discharge about aftercare and rehabilitation as unclear or incomplete.	
	Competency	Competent healthcare professionals	Patients described healthcare professionals as competent, experienced, and routinised, which resulted in feeling safe, secure and being in good hands.	“… That the caregivers are calm and show that they are knowledgeable, and you feel then that you’re in good hands" [Patient 27, male, age 55 years, five days admission to the hospital].
		Unfamiliarity with the virus	They had the feeling that healthcare professionals knew exactly what they were doing even though they were unfamiliar with the COVID-19 virus.	
		Inexperienced healthcare professionals	A few patients mentioned that they noticed that healthcare professionals were inexperienced which led to a sense of fear and feelings of uncertainty for recovery. At the same time, patients understood that it was a new situation with a lot of uncertainties and new protocols were not yet properly defined.	"It was just an uncertain thing and that was true for everyone lying there because they didn't know anything, the doctors couldn't say anything either, nobody knew anything." [Patient 11, male, age 52 years, three days of admission to the hospital]
	Physical care	Receiving help	Patients had positive experiences with physical care. They received help and support when necessary, e.g., with washing and getting dressed. Patients mentioned that they were encouraged to carry out physical care by themselves and to eat and drink sufficiently.	"That's so exactly what they did, that's what I mean by what they have to do, they do, but they try to make you do as much as possible yourself” [Patient 6, male, age 78 years, eight days of admission to the hospital].
		Regular checks	Healthcare professionals kept an eye on them by performing regular checks, e.g., by asking how the patient was feeling, checking their vital signs, and taking blood samples.	
		Not needing help with physical care	One-third of the patients mentioned that they did not need any help with physical care.	
Care environment	COVID-19 nursing ward	Stay in a multiple room	Patients experienced their stay in a multi-person room as positive.	“And everybody is in the same boat there, so in that respect that’s kind of a bonding thing ... we all have to do it and try to get everybody back on their feet I think” [Patient 19, female, age 54 years, five days of admission at the hospital].
		Stay in a single room	Patients experienced their stay in a single room as positive.	
		Contact with other patients	They experienced togetherness with other patients all being in the same situation and fighting the disease together.	
		Impact seeing other ill patients	Patients mentioned they felt no need to have contact with other patients. By seeing other ill patients, they became aware of the seriousness of the situation, compared themselves with others and felt that they were lucky their situation was improving while this was not the case for everyone.	“What you also realize very well is that people die there. I saw that too, we also saw someone die there ... that was very confronting that you realise you have a disease.” [Patient 17, female, age 48, four days of admission to the hospital].
	Isolation precautions	Impact of the care environment	In general, patients reported that isolation precautions were well organised.	“… you're in a ward with big doors and red ribbons … I did not feel locked in though, but I mean you were of course, very isolated from the rest of the world.” [Patient 2, male, age 72 years, three days of admission at the hospital].
		Isolated from the outside world	Patients experienced being isolated from the outside world, but a few patients mentioned being isolated gave rest.	
		Understanding the importance of isolation clothes	They understood and accepted the importance of wearing isolation clothes for self-protection.	
		Accepting the situation	Other patients mentioned they did not pay any attention to the isolation precautions, because they felt too ill to think about the situation.	
	No visitors allowed	Accepting ‘no visitors’	All patients accepted the situation that visitors were not allowed to enter the COVID-19 nursing ward. They mentioned that safety for everyone was important as they did not want to infect their loved ones, and sometimes even felt too ill to receive visitors.	“But yes if that possibility is not there, yes also to protect them because of course that is also very important, of course it is not only for my own interest, but also in their interest ...” [Patient 13, male, age 52 years, four days of admission].
		Receiving no visitors is difficult	However, some patients also mentioned that it was very difficult not to receive any visitors. They felt lonely and missed (physical) contact with their loved one(s) who they normally see daily.	“Yes, I found that a bit difficult. Or difficult, yes, there I did feel like: oh, oh, I would so like to hug him for a little while say hey.” [Patient 3, female, age 67 years, seven days of admission].
		Need for visiting opportunity	Patients felt the need to have visiting opportunities.	
	(Video)calling	(Video)calling positive experiences	Patients experienced contact with family by (video) call as positive and acceptable as a replacement for visits in this crisis. They were happy with the opportunity to see their family members by (video)call.	“The distance, the seeing, you see each other, you experience each other, you feel each other, and through the phone that’s not there. But, yes, you know it’s the way it is, so you deal with it.” [Patient 2, male, age 72 years, three days of admission at the hospital]
		(Video)calling limitations	However, patients experienced (video) calling as limited and inherently different from physical contact.	
		No need for (video)calling	About one-third of the patients described that they felt no need to have contact because they felt too ill and (video) calling was difficult or exhausting.	
	Isolation clothes	Impact of isolation clothes	Patients experienced that seeing healthcare professionals work in isolation clothes had a lot of impact, was impressive, unreal or strange. Several patients compared the situation with ‘being in outer space’.	“It’s like being on the moon, with all those people in all those suits...” [Patient 1, female, age 67 years, five days of admission at the hospital].
		(no) Impact of unrecognizability	Patients mentioned that healthcare professionals were unrecognisable due to their isolation clothing: only the eyes were visible, which was difficult or exhausting not knowing who was standing in front of them.	
		Contact with healthcare professionals (not) hampered	They mentioned that isolation clothing hampered the contact with healthcare professionals.	“It’s just a pity, but that couldn’t be helped, that doctors and nurses are quite rightly well wrapped up ... that did make the personal contact a little less, everyone seems the same with one of those mouth masks and goggles and a hat and a blue apron ...” [Patient 22, male, age 61 years, seven days of admission to the hospital]
		Attempt to improve identification	Additionally, patients mentioned it was pleasant that healthcare professionals wore nametags or photos on their isolation clothes to improve recognisability.	
Person-centred processes	Engagement	Motivation of healthcare professionals	Patients experienced healthcare professionals as engaged, motivated and were aware that professionals did their best to provide the best care by giving them attention.	“There were often moments when it just got too much and you were just crying and thinking how can this be? Yes, they would come and sit with you for a while and listen carefully. That just does you good ...” [Patient 19, female, age 54 years, five days admission at the hospital].
		Experiencing attention	Patients experienced caring and humanity in terms of a personal approach, they felt understood regarding their situation, and found that healthcare professionals took time to listen actively to them. Patients mentioned that this engagement led to the feeling of being heard, feeling safe, feeling connected, and fighting their disease together.	
		Distance in contact	In contrast, other patients mentioned distance and a task-oriented type of contact: they experienced little contact and insufficient personal attention from healthcare professionals.	
	Needs	(no) Attention for needs	Patients experienced that it was pleasant and comforting that healthcare professionals paid a lot of personal attention to their needs by spending time to talk to them and showing understanding of the situation they were in. In contrast, a few patients experienced that healthcare professionals did not connect to their needs.	“... I’ll come and sit with you in a moment ... Well and then they would just come and sit with you and yeah, I think: well these are, these are just much better pills than anything else ... the personal attention to the patient is so, so incredibly important.” [Patient 3, female, age 67 years, seven days admission to the hospital].
		Having no needs to attend to	A few patients felt they had no needs to attend to by healthcare professionals.	
		Healthcare professionals took initiative	Patients described that sometimes healthcare professionals took initiative themselves to fulfil patients’ needs. One patient appreciated that a care professional had a good idea to personalise the room of the patient.	“And that nurse had a brilliant idea: she said get your husband and children to send or mail me photos of them and the grandchildren, and then I’ll print them out and hang them up at the end of your bed. That gave me such a boost; it was really lovely that when I opened my eyes, there they all were” [Patient 3, female, age 67 years, seven days of admission to the hospital].
		Discharge from the COVID-19 nursing ward	Furthermore, a few patients mentioned they were discharged from the hospital too quickly because they were still feeling very ill.	
	Shared decision- making	(no) Involvement in decisions about treatment/care	Patients experienced involvement in decisions about the treatment and care they received positively. They were stimulated to participate in deciding, e.g., about treatment with oxygen if they felt they needed it, in participating in an experimental treatment with hydroxychloroquine, and decision-making about discharge. Relevant care issues were discussed, e.g., taking a shower or washing by the bed and the patient’s preferences were taken seriously. Sometimes, patients were active in the process and took initiative themselves to get involved. On the other hand, some patients experienced no involvement.	“Everything was discussed, and if I didn’t want certain things to be done, then they weren’t. I really felt that they listened to me” [Patient 3, female, age 67 years, seven days of admission to the hospital].
		Feeling no need for involvement	Some patients felt no need to be involved, or they felt too ill to decide.	“No, that wasn’t discussed, and nor should it have been because I really don’t know anything about it, but they do, so I think it’s only right that they don’t discuss it with the patient.” [Patient 2, male, age 72 years, three days admission at the hospital].
		Having no choice in treatment	Some patients felt that they had no choice in their treatment other than to resign to care due to their condition. Patients trusted healthcare professionals’ decisions about treatment or care as they did not have the knowledge about treatment themselves.	
Results of care	Recovery	Treatment/care/environment/healthcare professionals were supportive	Patients mentioned that they recovered with the help of the given care. For example, by medication, oxygen therapy, receiving physical help from healthcare professionals, food, receiving rest, and being stimulated to exercise.	“Yes, … I recovered. And I think that this was partly attributed to the received care and medications …” [Patient 18, male, age 50 years, two days of admission to the hospital]
		Being recovered	Patients mentioned that they were fully recovered from COVID-19 and some more quickly than expected.	
		Happy to be home	Patients were happy to be home.	
		Having residual symptoms	Other patients mentioned they experienced residual symptoms.	
		Better guidance at home is necessary	Patients mentioned that more or better guidance for recovery at home was necessary. They would have appreciated the possibility to call a care professional from the hospital if problems at home occurred.	

Domain 1: Healthcare Professionals’ Abilities

Three themes were identified: communication, competency, and physical care. Patients evaluated professionals’ abilities positively and showed their appreciation and gratitude towards healthcare professionals. Patients were aware of the difficult circumstances professionals worked in, in relation to the isolation precautions.

Domain 2: Care Environment

Five themes were identified: COVID-19 nursing ward, isolation precautions, no visitors allowed, (video)calling and isolation clothes. This domain was mostly affected by the pandemic. Patients reported business and a high rotation of healthcare professionals in the COVID-19 nursing ward. Isolation clothes hampered contact with healthcare professionals, although name/photo tags improved this. Patients understood the necessity for isolation precautions to protect others, but it did impact their wellbeing. Receiving no visitors was difficult and resulted in feeling lonely and missing physical contact. (Video)calling was experienced as an alternative to stay in contact with their relatives. However, it was no replacement for physical contact. Although some still felt isolated, the cohort department contributed to feelings of togetherness with other patients having the same illness.

Domain 3: Person-centred Processes

Three themes were identified: engagement, needs and shared decision-making. Patients were positive about healthcare professionals’ engagement and their attention to the patient’s needs. Patients felt limited need for interaction in shared decision-making because of feeling too ill or having not enough knowledge.

Domain 4: Results of Care

One theme was identified: recovery. Patients mentioned that they recovered with the help of the given care.

Differences between the acute and semi-acute phases

For the PREM questionnaire, on question 5, an inadequate experience with care was scored more often for the semi-acute phase: n=18 (30.5%) versus n=6 (11.5%) (p=0.003) (see Table 5). For the interviews, more patients in the acute phase mentioned they were satisfied with the communication with healthcare professionals (13 versus seven), friendly healthcare professionals responding to their needs (12 versus eight), and experienced isolation from the outside world (eight versus three). More patients in the semi-acute phase mentioned competent, experienced, and routinised healthcare professionals (12 versus eight), felt less isolated and experienced (video)calling as positive (10 versus six), and that healthcare professionals wore nametags or photos on their isolation clothes to improve recognisability (10 versus three).

**Table 5:** Categories of scores of PREM questions 1, 2 and 5 that differed for the acute phase and semi-acute phase. No differences were seen for questions 3, 4, 6 and 7. Note: PREM = Patient Reported Experience Measure, Q = question, n = number, NA = not applicable, P = probability, X^2^ = chi-square, df = degrees of freedom. ^1^Questions 1, 2, 4 and 5 categorised as inadequate (1-5), adequate (6-7), and good (8-10) experience with care, and NA. ^2^Chi-square test. ^3^Significant differences (<0.05) in categories between the acute and semi-acute phase.

Scores^1^	Q1				Q2				Q4				Q5				
	Acute phase (March) (n=59)	Semi-acute phase (April-June) (n=52)	P	Test statistics X^2^ (df)	Acute phase (March) (n=59)	Semi-acute phase (April-June) (n=52)	P	Test statistics X^2^ (df)	Acute phase (March) (n=59)	Semi-acute phase (April-June) (n=52)	P	Test statistics X^2^ (df)	Acute phase (March) (n=59)	Semi-acute phase (April-June) (n=52)	P	Test statistics X^2^ (df)	
	n (% of acute/semi-acute phase)				n (% of acute/semi-acute phase)				n (% of acute/semi-acute phase)				n (% of acute/semi-acute phase)				
1-5	0	0	0.040^2,3^	6.450 (2)	2 (3.4)	3 (5.8)	0.100^2,3^	6.255 (3)	8 (13.6)	5 (9.6)	0.349^2^	3.290 (3)	18 (30.5)^3^	6 (11.5)^3^	0.003^2,3^	14.028 (3)	
6-7	5 (8.5)	8 (15.4)			6 (10.2)	8 (15.4)			5 (8.5)	8 (15.4)			5 (8.5)	11 (21.2)			
8-10	48 (81.4)	44 (84.6)			45 (76.3)	41 (78.8)			32 (54.2)	32 (61.5)			26 (44.1)^3^	33 (63.5)^3^			
NA	6 (10.2)^3^	0^3^			6 (10.2)^3^	0^3^			14 (23.7)	7 (13.5)			10 (16.9)^3^	2 (3.8)^3^			

## Discussion

Statement of principal findings

We investigated the experiences and well-being of patients receiving care in cohort isolation during the acute and semi-acute phases of the first COVID-19 pandemic wave. In general, median scores on all PREM questions were high, although the range was widespread, indicating that not everyone was satisfied. In line with the PREM results, the interviews indicated that the majority of patients experienced ‘healthcare professional’s abilities’, ‘person-centred processes’ and ‘results of care’ positively. Patients understood that little was known about the new virus, and they adapted their expectations accordingly. In contrast to the interviews, the lowest scores on the PREM questionnaire were seen on shared decision-making. The ‘care environment’ was affected mostly and isolation precautions impacted patients’ well-being.

Integration and discussion of the results

The PREM scores on shared decision-making were lower despite positive feedback about this topic in the interviews. This might be explained by the fact that patients were asked in the PREM whether they were involved in the decision, and not whether they wanted to share in the decision-making. Some patients explained in the interviews they did not want to be involved because they felt too ill and had no knowledge about treatment or care, which is in contrast to another study [26] explaining that COVID-19 patients wanted to be more involved, although they experienced a lack of information provision about treatment decisions, which was not the case among the patients in our study. We believe that patients should get the choice if they want to be involved or leave the decisions to the professionals.

The ‘care environment’ was affected mostly and had a negative impact on patients’ well-being, mainly due to the isolation clothes. In accordance with previous studies [7,26,27], patients felt that seeing facial expression was very important to establish sufficient contact with healthcare professionals; this was now hampered by the masks. In line with Roderick et al. [28], patients were positive about using name and photo tags on isolation clothes in the semi-acute phase to improve contact.

Isolation precautions had negative effects on patients’ well-being: they felt lonely and missed physical contact with relatives. (Video)calling was of limited benefit and different from physical contact, which is in line with earlier findings regarding isolation during the outbreak of severe acute respiratory syndrome (SARS) in 2003 [12]. A recent study showed that patients without relatives’ support experienced higher depression levels during quarantine than patients who stayed in phone contact with relatives [27]. Therefore, family visits should be facilitated to prevent depression symptoms. In case of isolation precautions, the visitors should also be suitably protected by isolation clothing and other measures as applicable.

In line with earlier research, the interviews showed that admission at a multiple-occupancy room on the COVID-19 nursing ward resulted in patients experiencing togetherness with other patients [13,29]: cancer patients experienced the multiple-occupancy room positively [13] and for COVID-19 patients the multiple-occupancy room improved patients’ wellbeing [29]. In both situations, it created the opportunity for the interaction with other patients having the same illness and provided a distraction from their illness. However, as also seen in our study, sharing rooms also created awareness of the severity of the situation: seeing other critically ill patients had much impact [29]. Therefore, admission at a multiple-occupancy room in cohort isolation should be organised to stimulate togetherness, but it is important to be aware of the impact on other patients.

Patients experienced much personal attention and engagement with healthcare professionals despite the hectic situation in the hospital. Cohort isolation might had made important contributions to these feelings of engagement. For example, internal doors were left open and healthcare professionals continued to walk around in the ward (in isolation clothes), leading to more contact moments like patients without any isolation precautions. In contrast, patients in a single-room isolation perceived a lack of respect for their needs and preferences [8] and decreased healthcare professionals’ contact [11].

Patients experienced less isolation and more competent healthcare professionals in the semi-acute phase. Less isolation might be explained by: arranging (video)calling, reduced workload for healthcare professionals because of reduced patient admissions [12,13] and wearing name or photo tags for recognisability. More competent healthcare professionals might be explained by: getting more experienced working with the new virus and guidelines for isolation precautions along the way.

Implications for policy, practice and research

Important lessons can be learned for future care in isolation. Patients should be given the choice of whether they want to be involved in shared decision-making. Name and photo tags on isolation clothes improve contact between the patient and the healthcare professional. We recommend to arrange cohort isolation - when possible - depending on isolation indication and the patient’s severity of the disease. Avoiding single- room isolation improves patients’ person-centred experiences and well-being. In cohort isolation, patients feel less isolated and more engaged with others because internal doors can be left open. However, it is important to be aware of patients’ responses and impact on their well-being by seeing other - severely - ill people. Allowing visits from family to improve patients’ well-being is important and feasible if they are protected with isolation clothes too.

Strengths and limitations

This research has some limitations. It was conducted in a single hospital, which may limit generalisability. Also, patients were contacted six to eight weeks after discharge, so recall bias may be present. Phone call interviews may have limited non-verbal communication, but, on the other hand, the likelihood of bias by socially desirable answers was probably low because of the anonymity of phone call interviews.

The main strength is the mixed-method design incorporating a large number of in-depth interviews in a heterogenous patient group, enabling in-depth interpretation of the PREM questionnaire results. We think care experiences can only be fully evaluated after analysing qualitative data from in-depth interviews as well.

## Conclusions

Patients’ experiences with person-centred care were positive, also because patients adapted their preferences to the circumstances. Most negative experiences were related to the isolation precautions. For future care, family and friends should be allowed to visit the patient whenever possible, and cohort isolation should be organised instead of single-room isolation, improving the patient’s well-being. Furthermore, name and photo tags should be used to improve contact between patients and healthcare professionals.

## Appendices

**Table 6:** Supplementary: interview guide

Theme	Questions
Admission	You were admitted to the hospital with symptoms. Could you tell me how your admission went and how you experienced it?
Contact with health care providers (e.g., medical specialists, nurses and paramedics)	How was the contact between you and the care professionals? How would you describe the skills of care professionals (including professional, information provision, commitment/motivation, conversation skills)? To what extent were you involved (included/informed/ encouraged) to have a say in (e.g., treatment/medication) the care that was provided? (How did you perceive decisions made about your care within the care environment?)
Care environment	If applicable, how did you experience the COVID suspected ward? To what extent was the environment you were in supportive in recovering from your illness? How did you experience the hospital environment to which you were admitted? How did you experience the isolation measures, to which you were exposed?
Feelings during care moments	How was your well-being (mental and physical) during the care moments (How did you feel)? How was the involvement of the care professionals (social, empathy)? To what extent were your personal needs and your opinion taken into account? How did the contact with your loved ones go/how did you experience this? How did you experience the rules regarding the visitation arrangements? How did you experience the physical care provided by the care professionals (e.g., nutrition/care/night rest/medication/oxygen)?
Concluding questions on received care, consequences, discharge and tips	Overall, how did you experience the care in the hospital? (and the care provided by the care professionals?) What do you think was good, and what do you think was not good? What effects/results did you experience from the care obtained? How did your discharge procedure go? (e.g., the provision of information)? How did you experience this? What tips would you like to give the hospital?
